# Supplementary material for: Are carbon emissions trading and green financial instruments synergistic? -Comprehensive quantitative research based on content analysis
Source: PLoS One. 2024 Mar 7;19(3):e0298601. doi: 10.1371/journal.pone.0298601 (PMC10919601; doi:10.1371/journal.pone.0298601)
Supplement: S1 Data — (ZIP) [file pone.0298601.s001.zip › Data/地级市Z_F工作报告.zip!/─┌├╔╣┼/░═╤σ─╫╢√╩╨/└┤╫╘╣1⁄2╓┌║┼í╛┬φ┐╦╩2╛▌═°í┐_2017.docx]

各位代表：

　　现在，我代表市人民政府向大会报告工作，请予审议，并请市政协委员和列席会议的同志提出意见。

**一、2016年工作回顾**

　　刚刚过去的一年，在市委的坚强领导下，全市上下深入贯彻落实中央、自治区党委、政府各项决策部署，团结一心、攻坚克难，全面做好稳增长、促改革、调结构、惠民生等各项工作，经济社会保持平稳健康发展，实现了“十三五”良好开局。

　　（一）全力以赴稳增长，经济发展稳中向好。坚持稳中求进工作总基调，积极推进供给侧结构性改革，认真落实“三去一降一补”政策，经济运行总体保持了稳中有进、稳中提质的态势。预计地区生产总值完成930亿元，增长7%；固定资产投资完成733.6亿元，增长10.4%；社会消费品零售总额完成258亿元，增长10%；一般公共预算收入完成69.95亿元，增长6.4%；城乡居民人均可支配收入分别达到26259元、14557元，均增长8%。现代农牧业稳定发展，粮食总产达60亿斤以上，设施农业达26万亩。牧业年度牲畜饲养量达2278万只，其中肉羊饲养量2128万只，出栏1151万只，肉羊出栏量稳居全区第一，创建成为国家级番茄制品出口质量安全示范区。工业经济平稳运行，预计规模以上工业增加值增长7%左右。东立多晶硅、西沙德盖钼矿采选、振森纤维板、圣牧高科有机酸奶等项目建成，恒嘉蓝宝石、美洋洋肉羊加工等项目加快推进。大数据云计算产业开始起步，服务器装机能力达到1万台。现代服务业发展势头强劲。黄河河套文化旅游区建设成效初显，河套酒业、黄河三盛公水利风景区成为国家4A级旅游景区，富强村、联星村分别被评为“中国最美乡村”、“中国最美村镇”。全市接待游客425万人次、增长20.7%；实现旅游收入43亿元、增长22.2%。4个电子商务产业园区建成投运，电商企业达到1082家，交易额增长20.4%，五原、前旗和杭后分别被评为国家、自治区级电子商务进农村综合示范县。金融机构存贷款余额分别达到937.1亿元和759.4亿元，存贷比达到81%。

　　（二）突出重点抓改革，发展活力不断增强。习近平总书记嘱托的三项重点改革稳步推进，70%以上龙头企业与农牧民建立利益联结机制，国有林场改革启动，对蒙合作改革试点深入实施。供给侧结构性改革成效初显，落实简政放权、减税降费、电价优惠等政策，降低企业生产成本24.75亿元，商品房库存量消化168万平方米。草原确权试点工作圆满完成，土地承包经营权抵质押贷款破题。“放管服”改革不断深化，市本级行政审批事项精简幅度达85%。“五证合一”“一照一码”商事制度改革全面实施。财税、金融改革稳步推进，严格落实国家“营改增”试点和资源税改革政策，采用PPP模式引进社会资本32亿元。综合行政执法改革走在全区前列。公交改革破题起步，国有公交公司组建成立。市工商、质监机构合并，食品药品监督管理局单独设置，组建4个旗县区安全生产监管机构。扩权强县、城乡居民医保制度整合、机关事业单位养老保险制度改革有序推进。教育、科技、文化、卫计等领域改革取得新进展。发挥口岸优势，加强内外开放，甘其毛都口岸全年过货1390万吨。全市进出口总额完成21亿美元，其中农畜产品出口4.2亿美元，连续7年位居全区首位。

　　（三）统筹城乡强基础，发展环境进一步改善。修编完成城市总规，编制中心城区25个专项规划和59个苏木乡镇规划，总规、控规和专项规划实现全覆盖。中心城区和县城建设水平有了新提升，投资156亿元，实施了先锋新桥、河套大街西延等一批城镇道路、管网、老旧小区和背街巷道改造工程，新增园林绿地2100亩，中旗被列入国家第三批新型城镇化综合试点。美丽乡村建设成效显著，新建和整治村庄2354个，新修通村公路2300公里，“十个全覆盖”任务基本完成。林业生态建设完成90万亩，乌梁素海综合治理列入国家“十三五”规划，规划方案通过自治区政府批复，纳林湖、巴美湖晋升为国家级湿地公园。河套灌区节水改造、黄河防洪二期、396万亩土地整治、50万亩高标准农田建设等工程扎实推进，我市被评为全国国土资源节约集约模范市。京新高速巴彦淖尔段主线贯通，甘临一级公路加快建设，中旗通用机场通航运营。群众盼望多年的临赛公路收费站取消收费，为临河-陕坝同城化发展打通了“瓶颈”，群众拍手称赞。

　　（四）千方百计惠民生，社会事业全面进步。在财力紧张的情况下，各级财政用于民生支出达178.5亿元，占一般公共财政预算支出的75.7%。全面完成脱贫攻坚“军令状”确定的年度目标任务，22857名贫困人口稳定脱贫，中旗、杭后、五原、磴口实现区贫旗县摘帽。新增城镇就业1.28万人，农牧民转移就业15万人，创建国家和自治区级众创空间试点5个。城乡居民养老、低保、五保供养保障标准稳步提高。开工建设保障房1.5万套，累计争取棚户区改造贷款和奖补资金近140亿元，为9000多户居民解决房产证办理遗留问题。中旗、杭后通过国家义务教育均衡发展验收，85所学校创建为国家、自治区级足球特色学校。市医院晋升为三甲医院，入驻新址运营。全面两孩政策稳步落实。实施“健康快车”扶贫项目，为农牧民免费开展白内障手术1000多例。新增授权专利194件、院士专家工作站2个、国家高新技术企业5家，巴彦淖尔农业科技园区被认定为国家农业科技园区，成功培育出世界首例超细型体细胞克隆羊。组织举办“翰墨书长征”书法名家作品全国巡展、河套文化艺术节、国际马拉松赛、全球华人篮球邀请赛、龙舟赛、沿黄河公路自行车赛、第一届市少数民族运动会等文体赛事活动，成功承办宁蒙陕甘毗邻地区联席会议第十三届年会，巴彦淖尔知名度和影响力进一步扩大。传媒大厦建成启用。食品药品监管不断强化，受到全国人大《食品安全法》执法检查组的高度评价。安全生产和环境保护“双百日攻坚”行动成效明显，信访形势总体平稳。社会治安形势持续好转，刑事和治安案件分别下降7.5%、5.4%，民意民生警务工作得到公安部肯定，群众安全感和满意度明显增强。社会信用体系建设取得新成效，国家发改委发布《全国259个地级市信用监测报告》，我市位居全国第50名、全区第1名。“五城同创”扎实推进，成为全国双拥模范城。民族、宗教、老龄慈善、妇女儿童、残疾人等各项事业都取得新进步。

　　（五）转变作风提效能，政府自身建设不断加强。认真落实改进作风各项规定，扎实开展“两学一做”学习教育，各级干部主动担当、积极作为的劲头明显增强。严格执行国务院“约法三章”，党政机关公务用车制度改革基本完成。自觉接受人大依法监督和政协民主监督，政府班子主动征求人大、政协意见和建议，积极配合人大开展立法工作。办理人大代表建议128件，政协委员提案240件，办结率均为100%。“七五”普法全面启动，政府法制工作进一步加强。审计、监察工作力度加大，反腐倡廉建设深入推进。

　　各位代表，过去的一年，是我们全市上下同舟共济、共克时艰、经受了各种考验的一年，也是我们奋力拼搏、砥砺前行、为“十三五”发展蓄积能量的一年。今天的巴彦淖尔，迈上了新台阶，站在了新的起点上。这一年成绩的取得，是市委统揽全局、科学决策的结果，是市人大、市政协监督与支持的结果，更是全市各族干部群众共同努力、不懈奋斗的结果。在此，我代表市人民政府，向所有关心支持巴彦淖尔事业发展的同志们、朋友们，表示衷心的感谢！

　　在总结成绩的同时，我们也要清醒地看到发展中存在的困难和问题，主要表现在：经济下行压力依然较大，部分企业生产经营困难，农畜产品价格受市场影响出现波动；改革推动和创新引领发展的能力还不强，新产业、新业态、新商业模式发展不足；财政持续增收难度加大，收支矛盾突出；部分地区生态环境还需加大治理力度，安全生产监管水平有待进一步提升；群众关注的上学、就业、就医、物业管理等方面还存在工作不到位的地方；一些机关工作人员作风不严不实，落实难、办事难、效率低等情况依然存在，等等。对这些问题，我们将加倍努力，始终以民之所望为施政所向，通过扎扎实实的工作，寻求突破，力求解决。

**二、2017年工作总体安排**

　　今年是实施“十三五”规划的重要一年，是供给侧结构性改革的深化之年，我们将迎来党的十九大胜利召开和自治区成立70周年，做好政府工作意义重大、机遇难得。我们必须审时度势，保持信心、勇气和定力，才能在新一轮经济格局大调整、大变革中赢得先机、争取主动。我们必须要瞄准目标不动摇，心无旁骛抓发展，才能使市第四次党代会绘就的发展蓝图变为现实，使“绿色崛起、赛过江南”的战略目标和市委的各项部署落地生根、开花结果。

　　今年政府工作的总体要求是：深入学习贯彻习近平总书记系列重要讲话和党的十八大、十八届三中、四中、五中、六中全会和中央经济工作会议精神，全面落实自治区第十次党代会、十届二次全委会、市第四次党代会精神，牢固树立和贯彻落实新发展理念，促进新型工业化、信息化、城镇化、农牧业现代化、绿色化“五化”协同发展，坚定不移做好“水、绿、文化”三篇文章，着力抓好“五大基地”建设，牢牢守住“三条底线”，努力在稳增长、促改革、调结构、惠民生、防风险上取得新成效，在全面建成小康社会、实现“绿色崛起、赛过江南”的征程中谱写新篇章。

　　主要预期目标是：地区生产总值增长8%左右，固定资产投资增长12%左右，社会消费品零售总额增长10.5%左右，一般公共预算收入同口径增长6%左右，城乡常住居民人均可支配收入均增长8%左右。全面完成自治区下达的环境保护约束性指标。确定这些目标，充分考虑了我们面临的形势任务和基础条件，考虑了与全面建成小康社会和“十三五”规划目标相衔接，是立足实际、经过综合权衡后确定的。这些目标不是轻而易举就能实现的，需要我们坚持稳中求进工作总基调，自我加压，迎难而上，盯住目标，一步一个脚印向前迈进，力争“跳起来摘桃子”，完成得更好一些，向全市人民交上一份满意的答卷。

　　我们要重点抓好以下十个方面的工作：

　　（一）坚持投资拉动，切实增强发展后劲

　　牢固树立投资是促进经济增长“第一拉动力”、重大项目建设是推动区域发展“第一抓手”的理念，坚持不懈抓招商引资和重大项目建设，力争完成固定资产投资822亿元，实施亿元以上项目152个，为自治区成立70周年献礼。

　　突出抓好“七网”建设，在铁路、公路、航空、市政、水利、能源、通信等方面，实施一批重大项目。铁路网建设，加快推进包头至银川高铁、临河物流中心铁路专用线项目，争取9月份开工。公路网建设，G7高速巴彦淖尔段、青山至乌根高勒一级公路、临河沿黄一级公路九庄段建成通车。国道242甘临一级公路主体完工，新改建农牧区公路400公里。推进国道242临河黄河大桥及引线工程、国道335乌不浪口至乌根高勒、国道331乌珠尔至北银根、国道110临河至磴口段二改一、省道311建丰农场至建设段、省道215乌不浪口至新安、省道212甘其毛都至海流图一级公路、纳林湖旅游公路、乌梁素海环湖公路、河套文化旅游公路、总排干堤岸公路等项目建设。航空网建设，做好前旗、后旗、磴口通用机场建设前期工作，力争早日实施。市政网建设，加强城市道路、供水、排水、供热、供气等基础设施建设，新改建各类管网317.6公里，新增集中供热面积475万平方米，天然气入户6360户、入户率提升3个百分点。水利网建设，实施跨区域水权转让试点工程、黄河防洪二期、灌区节水改造、节水增效等项目，构建内外连通、河湖一体的城乡水网。能源网建设，推进电网、输气管道、运煤通道等设施建设，实施32项输变电工程。通信网建设，推进宽带巴彦淖尔建设，实施国家电信普遍服务试点，完成627个农垦农牧场和部分行政村、自然村宽带升级改造。

　　深入开展“项目建设年”活动，多方争取国家和自治区投资，充分激活民间投资，支撑保障经济发展。按照“干着今年、想着明年、备着后年”的工作思路，做实项目前期工作，认真落实重大项目三年滚动计划，谋划、储备、建设一批重大项目。认真组织好春季和秋季重大项目、重大工程集中开工推进活动。大力开展招商引资，发扬“四千”精神，在确保符合产业政策和有质量有效益的前提下，一二三产齐抓，大中小项目全上，央企地企、国企民企都招，实现招商引资项目大落地、总量大突破。加强项目建设调度，建立重大项目专项推进机制，加大考核力度，确保投资持续稳定增长。

　　优化营商环境，打造诚信规范、互利共赢、温馨包容、公平公正的投资环境，着力构建“亲”“清”政商关系，营造尊重和关爱企业家的社会氛围。放心、放胆、放宽、放活发展非公有制经济，充分激发企业家精神和工匠精神，支持本土企业做大做强，做成“百年老店”，鼓励在外地的巴彦淖尔企业家回乡创业。

　　（二）坚持农牧业强基，促进现代农牧业转型升级

　　农牧业是巴彦淖尔安身立命之本，必须紧紧抓在手上，做深做细做精。深入推进农牧业供给侧结构性改革，充分发挥资源优势，大力发展绿色、生态、节水、高效农牧业，加快形成结构合理、保障有力、发展可持续的农畜产品有效供给，当好全区绿色农畜产品生产加工基地排头兵。

　　调优农牧业结构。农作物总播面积稳定在1000万亩以上，粮食产量保持稳定。大力发展高效特色种植业，扩大青贮和优质牧草种植面积，新增设施农业2.5万亩，建成临河乌兰图克、五原八里桥2个万亩以上设施农业园区。优质小麦种植面积达到150万亩以上。通过引进龙头企业，优化畜牧业结构，稳羊增牛、扩大猪禽、适度发展肉驴和水产等特色养殖，肉羊饲养量达到2400万只。

　　提升农牧业发展保障能力。完善基础设施建设，启动实施480万亩改盐增粮增草工程，完成50万亩高标准农田、20万亩农机深松整地和土地整治等项目。推动发展土地托管、联耕联种、股份合作等多种经营模式，发挥农机合作社、农牧民专业合作社组织作用，实施国家新型经营主体带头人轮训计划，多路径提升规模经营水平。推进现代种业建设，做好专用小麦、青贮玉米、杂交向日葵、加工型蔬菜的新品种选育，开展巴美肉羊新品系选育和肉牛等胚胎移植。健全疫病防控、标准化生产等社会化服务体系。开展质量提升行动，加强全面质量管理，健全完善农畜产品监管、检测、执法体系。开展万名师资团培训，完成农牧民科技培训30万人次。举全市之力，建设国家农业高新技术产业示范区。

　　扎实推进品牌强市战略，挖掘培育具有地方特色的著名商标和名牌产品，建立梯次合理的品牌资源后备库。加大绿色农畜产品品牌建设力度。围绕“河套”“巴彦淖尔”地域品牌，做好“三品一标”产地认定和产品认证，申报河套小麦、磴口华莱士、乌拉特黑山羊等国家地理标志产品登记保护，让我市农牧业“金字招牌”更亮。

　　推进农牧业与二三产业融合发展。巩固提升农牧业产业化水平，拓展延伸农牧业生态保护、文化传承、旅游观光、休闲度假、养生养老等功能，大力发展观光农牧业、体验农牧业、科普农牧业，提高农牧业全产业链收益。

　　（三）坚持工业增效，推动工业经济稳中有进

　　坚持发挥优势和补齐短板一起做、盘活存量和做优增量同步抓，推动产业向绿色化、高端化、智能化、多元化方向发展，落实好“三去一降一补”政策，促进工业经济提质增效。全年力争完成工业投资378亿元，实施千万元以上工业项目146项。规模以上工业增加值增长7.6%。

　　加快传统产业改造升级。运用高新技术和先进适用技术，改造提升农畜产品加工、矿山冶金、能源电力、化工等传统支柱产业。农畜产品加工方面，加快推进润海源8000万只肉鸡加工、康斯特4500吨低酯果胶、海成生物肠粘膜蛋白和肝素钠提取等项目建设。支持羊绒、面粉、酿酒、乳品等企业实施技改，培育壮大农畜产品加工领军企业。推动中粮集团与恒丰公司开展合作。矿山冶金、电力和化工方面，开工建设华拓矿业节能技改、铸合800万吨银矿采选、海钰硫磷钛铁循环利用、青园科技12万吨可降解地膜等项目，加快推动20万吨铜冶炼、神华一期填平补齐等项目，延长煤化工、有色和黑色金属冶炼等产业链条，提高产品附加值。深化与包钢集团合作，加快启动10万吨甲醇合成油、8万吨苯加氢、100万吨低温半焦等项目。

　　大力发展新兴产业。安排新兴产业引导专项资金和小微企业技术进步贴息资金，加快培育新能源、新材料、大数据、生物科技等新兴产业。开工建设盾安5000吨、东立6000吨多晶硅项目，推动恒嘉800吨蓝宝石、中旗百万千瓦风电基地、沙漠生态光伏发电等项目建设。积极推进与全国、全区石墨产业发展联盟协作，建设石墨高新技术产业园，推动石墨新材料产业发展。坚持以设施为基础、以安全为前提、以资源为根本、以应用为核心的思路，培育发展大数据产业，推进智慧巴彦淖尔、农畜产品加工、农畜产品质量追溯、水资源管理、电子商务等平台建设。

　　加强工业经济运行调度。认真落实中央和自治区扶持企业发展的政策措施，加强对全市重点工业产品价格、用电负荷、产品库存监测，做好煤电油运等要素保障，帮助企业稳定生产经营，促进符合产业政策的企业恢复生产。

　　增强园区集聚发展能力。立足园区产业定位，围绕优势产业项目，培育发展循环经济，建设园中园、特色园，提高产业集中度和集群化水平，做大园区产业规模。力争产值超百亿元园区达到5个，产值超10亿元企业达到20家，紫金公司、联邦制药、圣牧高科产值突破50亿元。

　　（四）坚持三产扩容，培育发展新的动能

　　坚持把服务业作为产业转型升级、拉动经济增长的重要动力，抓好旅游、金融、物流、电子商务等产业发展。

　　提升旅游业发展水平。实施“旅游+”战略，着眼发展全域旅游、四季旅游，实现美丽全覆盖。完善全市旅游业发展规划。加快黄河河套文化旅游区建设，完成酒庄老镇、黄河古渡、观凌塔等项目，力争创建国家5A级旅游景区。推进乌梁素海、纳林湖、沙海旅游区、三盛公黄河渔村等景区建设。坚持以农兴旅、以旅促农，扩大休闲农业与乡村旅游建设面，把村庄变景点，村居变客房，土产变商品，积极培育发展乡村旅游。充实旅游文化内涵，对丰富的历史文化资源进行创意化开发，凸显旅游的文化性和体验性，提升旅游与文化融合度。大力发展冬季旅游，举办首届河套冬季文化旅游节、花灯会等活动，让冬季旅游火起来。加大旅游宣传推介力度，推动旅游区域合作。办好国际驼球大赛、沿黄河公路自行车赛、河套湿地龙舟赛等大型赛事，办好中国羊业大会、农畜产品博览会、河套旅游推介会。全年接待游客500万人次以上，旅游总收入突破50亿元。我们要把旅游业培育成经济转型升级重要推动力、生态文明建设重要引领产业、展示地区综合实力的重要载体、打赢脱贫攻坚战的重要生力军，为实现“绿色崛起、赛过江南”作出重要贡献。

　　大力发展金融业。拓展普惠金融，提高金融服务的覆盖率和满意度，让群众获取价格合理、便捷安全的金融服务。完善金融组织体系，积极引进各类金融机构，发展小微金融机构和准金融机构，鼓励民间资本引进参股投资或发起设立各类金融组织。发挥市城投、农投、交投、水投等融资平台作用，适时组建旅游投资平台。坚持兜住底线、把控风险、严格程序的原则，推广运用PPP模式，激发社会投资活力。力争信贷总量达到1100亿元以上，社会融资规模增量达到200亿元。完成企业上市三年行动计划，新增23家企业进入多层次资本市场，直接融资50亿元以上。

　　加快电子商务和物流业发展。继续推进电子商务示范县、示范基地、示范企业建设。扩大羊畜产品交易中心、渤海商品交易所五原葵花籽交易中心交易规模，筹建华夏非物质文化遗产交易和大宗农畜产品交易平台，形成市场定价权。支持巴团网、河套购等本土第三方服务平台发展，坚持“线上”与“线下”相结合，深化与阿里巴巴、京东等电商企业合作，推动我市名优特农畜产品进军全国、全球市场。举办农村电商巴彦淖尔峰会，规范发展物流快递业，支持巴运物流等企业发展，加大快递、网购下乡进村力度。

　　（五）坚持新型城镇化战略，提高宜居宜业水平

　　扎实推进以人为核心的新型城镇化，牢固树立民生城建理念，坚持生产生活生态相融，扎实推进“五城同创”，科学编制城市规划，完善城镇功能，改善人居环境，让群众生活得更方便、更舒心、更美好。

　　加大城镇基础设施建设力度，完成城建投资160亿元，重点实施五大工程。一是平房区和老旧小区综合改造工程。改造老旧小区90万平方米、平房区52万平方米，其中临河分别完成60万平方米、30万平方米改造任务。二是空气环境质量改善工程。实施“蓝天”行动，全市新增平房区集中供热面积61万平方米，其中临河50万平方米，撤并全部燃煤小锅炉，为5000户平房区居民免费换装环保节能炉具。完成自治区下达的淘汰黄标车和老旧车任务，抓好冬季雾霾和重点行业烟粉尘治理，争取PM2.5浓度下降3.5%，全年优良天气达到300天以上。三是居民饮用水保障工程。完成黄河水厂续建及配套工程，日供水能力达到10万吨，让群众喝上优质的黄河水。四是城市道路畅通工程。全市新改建城镇道路91.2公里、背街巷道59.4万平方米，完成金川大道北出口、市区至青春湖景区西出口、前旗西出口、中旗南出口等15个出入口改造，打通影响群众出行的河套大街、利民街、庆丰街、朔方路、明珠南路、丰州北路、磴口黄河西街、五原团结西路等“断头路”，让群众出行更加方便。五是园林绿化提档升级工程。坚持适地适树，优选抗病虫、耐盐碱树种，乔灌结合，花草搭配，四季见绿，提高绿化的层次感和色彩感。新建足球主题公园、水景公园、富源公园等公园游园，新增城镇园林绿地1800亩，高标准建设临河植物园综合工程，创建国家园林城市，启动“森林城市”建设。

　　优先发展中心城区。坚持市区共建，统筹地上地下、新区老区，在资源配置、项目布局、资金投入等方面给予倾斜支持，着力提升临河区辐射带动能力。按照建设城市草原、黄河外滩、中国西部最美生态休闲旅游目的地发展定位，坚持“三分之一水、三分之一绿、三分之一公建和新兴产业”的规划布局，加快双河新区开发建设，完成控制性规划修编。完成先锋新桥、先锋南路、云中大街、敕勒大街建设，河套学院新校区建成投用，打通南出口与沿黄公路连接线，形成10平方公里核心区主框架。推进市委党校新校区、内蒙古美术职业学院、电力综合服务中心建设。推动临河-陕坝同城化发展。围绕高铁线位布局和站址设置，优化沿途及周边城镇规划建设。积极发展高铁经济。

　　加强城市精细化管理。围绕“整洁畅通有序”的目标，着力解决城市公共交通、物业管理、集中供暖、环境卫生等一批群众关注的实际问题。完善土地储备制度，依法清理盘活闲置土地。增强广大市民文明意识，形成共建、共管、共享的城市发展氛围，提升全社会文明水平。做好全国文明城市创建各项工作。

　　建设美丽村镇。抓好23个重点镇建设，积极打造特色小镇。加大农村牧区环境突出问题综合治理力度，配套完善污水收集、垃圾处理等设施。健全村庄管护长效机制，让干净整洁、环境优美的农牧区成为巴彦淖尔的亮丽风景。

　　（六）坚持生态优先，着力保护好绿水青山

　　生态就是资源，美丽就是资本。我们要像保护眼睛一样保护生态。

　　加大湿地保护与恢复力度。按照“生态补水，控源减污，修复治理，资源利用，持续发展”的思路，加快乌梁素海综合治理，完成补水2.5亿立方米以上，争取治理规划获得国家批复。36个水质监测点全部建成投用，实现乌梁素海上下游污染源监测全覆盖。实施多蓝湖、青春湖、章嘉庙湖、金川河北延、杭后北郊湿地等工程，完成永清河（北边渠）综合治理2.5公里，改造中旗西河槽、后旗迎宾湖等湿地和水系工程，恢复湖泊湿地15处，实现“一旗县恢复一湖（湿地）”的目标，全市恢复湿地和湖泊3万亩。实施沿黄公路防洪堤加固及周边生态保护工程。稳步落实“河长制”，分级分段设立河长，解决好河湖管理突出问题，维护河湖生态功能。

　　推进重点生态区域治理。加大退耕还林还草力度，实施京津风沙源治理、天然林保护等工程，推进“森林机场”、主要通道、村庄、农田防护林、城镇出入口等重点区域绿化，完成林业生态建设90万亩，新增经济林2.2万亩。巩固生态建设成果，加强病虫害防治，做到有灾不扩散。落实新一轮草原生态补奖政策，推进乌兰布和沙漠治理，新增肉苁蓉、酿酒葡萄、黑枸杞等沙产业1万亩。

　　加强突出环境问题治理。全面整顿和规范矿产资源开发秩序，推进矿山地质环境恢复治理，开展乌拉山、乌兰忽洞矿区、采选企业尾矿坝等重点区域治理。抓好各类自然保护区建设和管理。深入实施化肥农药使用零增长行动，推进秸秆、地膜等农业废弃物资源化利用和畜禽养殖污染治理。抓好中央环保督查反馈问题整改，解决好群众反映强烈的环境问题。我们要持续用力、久久为功，努力建设全国生态文明先行示范区，让巴彦淖尔的天更蓝、地更绿、水更清、空气更清新。

　　（七）坚持民生为本，切实提升群众幸福指数

　　幸福安康是民心所向。要守住民生底线，多办顺民意、解民忧、增民利的好事实事，让群众共享改革发展成果。

　　打好脱贫攻坚战。把脱贫攻坚作为全面建成小康社会的重中之重，作为头等大事和第一民生工程，深入贯彻“精准扶贫、精准脱贫”基本方略，认真落实“五个一批、六个精准”要求，加大扶贫开发投入力度，形成外部多元扶贫与内部自我脱贫的互动机制，确保14171名贫困人口全部脱贫，前旗实现脱贫摘帽，精准发力，持续用力，以超常规举措打赢脱贫攻坚战。

　　积极扩大就业。提高技能培训针对性和就业率，推进创业园建设，落实创业担保贷款政策。新增城镇就业1.25万人，农村牧区劳动力转移就业14.5万人，启动市职业技能培训基地和示范性综合实践基地建设。大力发展众创、众包、众扶、众筹等新模式，支持分享经济发展，掀起大众创业、万众创新的热潮，努力让人人都有干事创业、实现梦想、人生出彩的机会。

　　提高社会保障水平。落实全民参保计划，扩大社会保险覆盖面，将更多进城务工农牧民、灵活就业人员纳入职工养老保险。扩大城乡低保覆盖面，新增4000名贫困农牧民纳入低保范围。做好残疾人生活、就业保障工作。加大困难家庭大病救助力度，建成市综合老年养护院。开工建设城市棚户区改造5000套，基本建成3169套。

　　办好人民满意教育。启动第三期学前教育三年行动计划，抓好苏木乡镇标准化中心幼儿园建设。完成义务教育标准化学校建设，临河、前旗、五原通过国家义务教育均衡发展验收。完成市直民族教育园区建设。支持和规范民办教育发展，提高继续教育和特殊教育办学水平。启动实施教育强县工程。

　　深入实施创新驱动发展战略。大力弘扬创新文化，调动全社会创新创业积极性。抓好科技创新平台载体建设，推进科技企业孵化器和众创空间建设，促进科技成果转化。加大人才培养引进力度，力争在医疗、教育、科研等方面引进更多高层次人才。

　　推进健康巴彦淖尔建设。大力发展蒙中医药事业，加强人才培养，实施“蒙中医药进万家”活动。开展医养结合试点，推进家庭医生签约服务，重点人群签约服务率达到100%。加强出生缺陷综合防治，提高出生人口素质。做好公共卫生和重大疾病防控工作，深入开展爱国卫生运动。

　　推动文化繁荣发展。进一步加强文化阵地建设，繁荣文化创作，推出大型纪录片《鸿雁》等一批文艺作品。加快培育新型文化业态，扶持和引导小微文化企业发展。支持和组织各类大型文化活动，举办河套文化艺术节，加快中蒙俄岩刻（画）文化长廊建设，办好中蒙俄国际岩刻艺术作品“双年展”、中蒙文化交流周等活动。做好阴山岩刻（画）申遗工作。促进体育事业发展，每个旗县区至少建设一个标准化足球场，每所学校建成1个足球场。强化舆论正面宣传引导，促进传统媒体与新兴媒体融合发展，提高巴彦淖尔新闻网、“掌上巴彦淖尔”“河套微传媒”等新媒体影响力和传播力，要把巴彦淖尔的故事讲得更美，名气传得更远。尽管财政增收的压力很大，但再紧再难，该给群众办的实事一件也不能少，我们要努力让群众的生活年年有新变化，一年更比一年好。

　　（八）坚持改革先导，全方位扩大对外开放

　　改革增动力，开放强活力。我们要始终把改革开放贯穿发展全过程、各领域，加快形成改革开放新优势。

　　加强供给侧结构性改革。打好降低企业成本“组合拳”，通过税费减免、电力扶持、金融信贷等措施，切实减轻企业负担。坚持“房子是用来住的，不是用来炒的”原则，有序推进房地产业发展，合理控制商品房增量，积极引导刚性购房需求和改善型购房需求，推进棚改货币化安置，消化房地产库存200万平方米。加强金融监管和监测预警，防范和稳妥化解金融风险。

　　深入抓好习近平总书记嘱托的三项改革。推广紧密型农企利益联结机制，产业化项目资金向利益联结机制好的企业倾斜，让农牧民更多分享产业化发展的收益。做好耕地、基本草原红线划定，制定森林资源有偿使用办法。深化对蒙务实合作，巩固双边定期会晤和警务工作机制，广泛开展中蒙教育、医疗、文体、旅游等人文交流，推进甘其毛都口岸“三互”大通关改革试点，实现联检部门联合监控、信息共享、协同执法。

　　深入推进重点领域改革。深化“放管服”改革，推行房地产和工业项目并联审批机制，实施全程电子化招投标和信用信息管理制度。全面推开“双随机一公开”监管制度改革，加快工商登记全程电子化和简易注销登记。深化财税体制改革，扩大国库集中支付范围，实现财政投资500万元以上项目全部跟踪评审。完成土地确权登记颁证工作，推进农村土地承包经营权、宅基地使用权、林权抵押贷款试点工作，积极探索农村承包土地“三权分置”改革。稳步推进农林“小三场”改革，完成国有林场改革，推进河套灌区水管体制改革。全面推进城市公立医院综合改革，建立薪酬制度和医疗绩效评价机制。积极推进行政综合执法改革，稳步实施事业单位、社会征信制度、社会治理体制改革。深化社会事业领域改革，确保各项改革取得实效。

　　提升对外开放水平。积极参与和服务“一带一路”、中蒙俄经济走廊建设，统筹口岸、陆港、保税物流中心建设发展，实现统一规划、功能互补、错位发展，形成多渠道进出口开放格局。抓好巴彦淖尔陆港建设，加快B型保税物流中心和铁路港申报，力争开通中欧农产品班列。申报建设甘其毛都口岸边境经济合作区，启动建设自治区级重点开发开放试验区、互市贸易区、跨境铁路、重载公路、通道改扩建、封闭式仓储等基础设施，提高通关查验、仓储物流能力，口岸过货量保持在千万吨以上。加强口岸城镇建设和管理，治理口岸及周边生态环境，筹划建设国门等标志性建筑。大力整顿口岸贸易秩序，支持神华等企业与蒙方开展矿能合作，推动境外煤铜资源落地加工、转化增值。深化区域经济协作，加强与京津冀、长三角、珠三角、宁陕甘等地区合作，深度融入呼包鄂经济圈和呼包银榆经济区，积极承接发达地区产业转移，努力实现共赢发展。

　　（九）坚持依法治市，维护和谐稳定环境

　　法治是社会稳定的“压舱石”，是实现社会公平正义的根本途径。我们要深入推进科学立法、严格执法、公正司法、全民守法，努力建设法治巴彦淖尔。

　　加强法治政府建设。严格按照法定权限、法定程序和权责清单行使权力，把政府活动全面纳入法治轨道。积极配合市人大常委会出台巴彦淖尔市黄河灌区水利工程管理保护条例，开展乌梁素海水环境保护立法。支持人民法院、人民检察院依法独立行使职权，努力让人民群众在每一个司法案件中都感受到公平正义。运用法治思维和法治方式解决社会问题、化解社会矛盾、规范信访秩序，提高律师、公证、法律援助等法律服务水平，发挥人民调解组织作用，维护群众合法权益。

　　加大安全生产监管力度。严格落实安全生产责任制，完善安全隐患数据库、安全监管专家人才库，建立健全第三方评估机制、安监部门信息沟通反馈机制，加大重点领域隐患排查和专项整治力度，坚决防止重特大安全事故发生。严格落实“四个最严”要求，严守食品药品安全监管的每一道防线，扎实推进国家食品安全城市创建，让群众吃得安全、放心。

　　加强和创新社会治理。推进以信息化、网络化为支撑的社会治安防控体系建设，依法严厉打击涉恐涉毒涉赌、金融诈骗、非法集资等违法犯罪活动，提升边境管控水平，确保“五个坚决不发生”。全面贯彻党的民族政策，深入开展全国民族团结进步示范市创建，推进兴边富民行动，重视做好城市民族工作，让“三个离不开”“五个认同”的思想深深扎根各族人民心中，促进各民族共同团结奋斗。依法管理宗教事务，积极开展双拥共建，支持国防和军队建设，巩固社会稳定、民族团结、边疆安宁的良好局面。

　　（十）坚持加强自身建设，建设人民满意政府

　　重任千钧惟担当。我们要把实现“绿色崛起、赛过江南”的使命扛在肩上，把万家忧乐放在心头，克己奉公，履职尽责，当好人民公仆。

　　严格遵守党的政治纪律和政治规矩，不断增强“四个意识”特别是核心意识、看齐意识，思想上衷心拥护核心，政治上坚决维护核心，组织上自觉服从核心，行动上始终紧跟核心，坚定自觉地同以习近平同志为核心的党中央保持高度一致。严格政府系统党内政治生活，自觉接受党内监督、社会监督、舆论监督，按照党章党规党纪履职尽责。认真落实市委的各项决策部署，自觉接受市人大及其常委会的法律监督、市政协的民主监督，认真听取人大代表、政协委员和各民主党派、工商联、无党派人士的意见建议。

　　全面推行政务公开，不断完善政府新闻发布会制度，扩大政务信息公开范围，对与群众利益密切相关的食品药品安全、保障房分配、事业单位公开招聘等信息，全部公开公示。推广电子政务和网上办事，抓好网上办事大厅、电子招投标平台建设。加强苏木乡镇政府和城市社区服务能力建设，创新服务方式，提高办事效率，打通服务群众“最后一公里”。

　　加强对重点领域、重点项目、重点资金的审计监督，强化行政监察，坚决整治行政不作为、乱作为和损害群众利益的行为。强化党风廉政建设，严肃查处违纪违法案件。严格落实改进作风各项规定，认真执行国务院“约法三章”。抓住想干事、敢干事这两个关键点，坚持以“实在实干实绩”为标杆，健全正向激励机制和容错纠错机制，倡导马上就办、夙夜在公、举轻若重、严守规矩、守土有责、勇于担当的工作作风。各级政府工作人员要进一步解放思想、革故鼎新，树立强烈的学习意识，努力提高本领素质，上上下下都要开动脑筋想工作，撸起袖子干事业，增强政府执行力和工作效率，在全市上下营造万众一心抓发展的浓厚氛围。

　　各位代表，实现“绿色崛起、赛过江南”的征程已经起航。让我们更加紧密地团结在以习近平同志为核心的党中央周围，在自治区党委、政府和市委的坚强领导下，守望相助，团结奋斗，一往无前，加快建设富裕文明绿色幸福巴彦淖尔，以优异的成绩迎接自治区成立70周年和党的十九大胜利召开！
